# Molecular genetics of cocaine use disorders in humans

**DOI:** 10.1038/s41380-021-01256-1

**Published:** 2021-08-27

**Authors:** Noèlia Fernàndez-Castillo, Judit Cabana-Domínguez, Roser Corominas, Bru Cormand

**Affiliations:** 1 Departament de Genètica, Microbiologia i Estadística, Facultat de Biologia, Universitat de Barcelona, Barcelona, Catalonia Spain; 2 Centro de Investigación Biomédica en Red de Enfermedades Raras (CIBERER), Instituto de Salud Carlos III, Madrid, Spain; 3 Institut de Biomedicina de la Universitat de Barcelona (IBUB), Barcelona, Catalonia Spain; 4 Institut de Recerca Sant Joan de Déu (IR-SJD), Esplugues de Llobregat, Catalonia Spain

**Keywords:** Addiction, Genetics

## Abstract

Drug addiction, one of the major health problems worldwide, is characterized by the loss of control in drug intake, craving, and withdrawal. At the individual level, drugs of abuse produce serious consequences on health and have a negative impact on the family environment and on interpersonal and work relationships. At a wider scale, they have significant socio-economic and public health consequences and they cause delinquency and citizen insecurity. Cocaine, a psychostimulant substance, is one of the most used illicit drugs, especially in America, Western Europe, and Australia. Cocaine use disorders (CUD) are complex multifactorial conditions driven by both genetic and environmental influences. Importantly, not all people who use cocaine develop CUD, and this is due, at least in part, to biological factors that are encoded in the genome of individuals. Acute and repeated use of cocaine induces epigenetic and gene expression changes responsible for the neuronal adaptations and the remodeling of brain circuits that lead to the transition from use to abuse or dependence. The purpose of this review is to delineate such factors, which should eventually help to understand the inter-individual variability in the susceptibility to cocaine addiction. Heritability estimates for CUD are high and genetic risk factors for cocaine addiction have been investigated by candidate gene association studies (CGAS) and genome-wide association studies (GWAS), reviewed here. Also, the high comorbidity that exists between CUD and several other psychiatric disorders is well known and includes phenotypes like schizophrenia, aggression, antisocial or risk-taking behaviors. Such comorbidities are associated with a worse lifetime trajectory, and here we report shared genetic factors that may contribute to them. Gene expression changes and epigenetic modifications induced by cocaine use and chronic abuse in humans are addressed by reviewing transcriptomic studies performed on neuronal cells and on *postmortem* brains. We report some genes which expression is altered by cocaine that also bear genetic risk variants for the disorder. Finally, we have a glance to the pharmacogenetics of CUD treatments, still in early stages. A better understanding of the genetic underpinnings of CUD will foster the search of effective treatments and help to move forward to personalized medicine.

## Clinical definition

The term substance use disorders (SUD), including cocaine use disorders (CUD), refers to different types of behaviors that range from sporadic use to abuse, dependence or addiction. The differential clinical diagnosis is based on the Diagnostic and Statistical Manual of Mental Disorders (DSM), which current version is DSM-5 [1], although many studies still use the previous DSM-IV-TR [2]. The main change of DSM-5 with respect to the previous version of the manual is the unification of abuse and dependence into a unidimensional category, SUD, that is qualified on a severity scale (i.e. mild, moderate and severe addiction) based on the number of symptoms endorsed, over a total of 10 (Table 1). In addition, DSM-5 drops one of the diagnostic criteria (legal problems) due to infrequent endorsement and poor discriminant validity [3], and adds a new one: craving. The World Health Organization developed another diagnostic interview, the International Classification of Diseases (ICD-11; https://icd.who.int/en), that keeps substance dependence as the main diagnostic criterion. Several authors have attempted to establish more homogeneous subgroups of cocaine-related phenotypes that may be useful for subsequent genetic analyses. Thus, five subtypes of cocaine abusers have been defined on the basis of clinical presentation, family history, and response to treatment [4]. More recently, cluster analysis was not only used to classify individuals on different groups according to cocaine-related measures but also to demographic features and prevalence rates of comorbid substance use and psychiatric disorders. Interestingly, those clusters characterized by a more severe phenotype yielded higher heritability estimates [5, 6].

**Table 1:** DSM-IV and DSM-5 criteria for Substance Use Disorders (adapted from Hasin et al. [1]).

	DSM-IV Abuse	Required criteria^a,b^	DSM-IV dependence	Required criteria^a^	DSM-5 substance use disorders	Required criteria^a^
Hazardous use	X	≥ 1	–		X	2-3 mild addiction
Social/interpersonal problems related to use	X		–		X	
Legal problems	X		–		–	
Withdrawal	X		–		X	
Tolerance	–		X	≥ 3	X	4-5 moderate addiction
Used larger amounts/longer	–		X		X	
Repeated attempts to quit/control use	–		X		X	
Much time spent using	–		X		X	≥6 severe addiction
Physical/psychological problems related to use	–		X		X	
Activities given up to use	–		X		X	
Craving	–		–		X	

^a^Within a 12-month period.

^b^A non-dependence diagnosis is needed.

## Epidemiology

According to the United Nations Office on Drugs and Crime, the number of people aged 15–64 years that use illegal drugs increased from ~210 to ~269 million over the period 2009–2018 (more than 28%, partly as a result of the global population growth) and the prevalence raised from 4.8 to 5.4% (12% increase) [7]. From these, over 35 million people suffer from SUD. Cocaine, together with metamphetamines, dominates the psychostimulants share. Some 19 million people used cocaine in 2018 (0.4% of the global population aged 15–64), fueled by the drug’s popularity in North and South America, Western Europe, and Australia. In Europe, cocaine is the most used illegal psychostimulant, around 5.4% of adults have tried cocaine [8], but at most 20% of them will develop addiction [9, 10]. From these data, the prevalence of cocaine dependence in the European population can be estimated around 1.1%, a value similar to that observed in American populations [11]. The prevalence of CUD is higher in males than in females, with 85% of cocaine users that initiate a treatment being males in contrast to 15% of females [8]. Ethnicity is also relevant, for example with rates of cocaine overdose deaths being much higher in African–American (AA) individuals in the United States [12]. For different reasons, research studies, and clinical trials have underrepresented women and ethnic minorities. This may lead to gaps in the discovery of risk factors for addiction, as several factors that influence the origin and development of the disorder differ across gender and ethnic groups. These factors include psychiatric comorbidities, sociodemographic status, frequency and severity of cocaine use, and also biological determinants [13, 14].

## Neurobiology

At the molecular level, cocaine binds the monoaminergic transporters (DAT, NET, and SERT) blocking the reuptake of these neurotransmitters by the presynaptic neuron, increasing the levels of dopamine, serotonin, and noradrenaline at the synaptic cleft [15]. Cocaine pleasurable and rewarding effects are mediated mainly by the increase of dopamine activity in the limbic system. Chronic cocaine use induces alterations and adaptations in several neurotransmitter systems and affects the function of several circuits and areas such as the mesocorticolimbic system (including the nucleus accumbens (NAc) and ventral tegmental area as well as prefrontal cortex). Serotonin neurotransmission is also key to cocaine addiction since it contributes to relapse by modulating impulsivity and responsivity to cocaine-associated stimuli [16].

Preventing relapse is the main challenge for treating CUD and SUD in general. Learning and memory processes associate memories between cocaine’s reinforcing and rewarding effects and environmental stimuli, which then will trigger cocaine craving during abstinence. This vulnerability to relapse, even after a long period of abstinence, involves stable gene expression changes and epigenetic modifications, especially in the corticostriatolimbic circuitry (hippocampus, prefrontal cortex, NAc, dorsal striatum, and amygdala) [17]. Stress plays an important role in relapse since increases drug craving, involving mainly the HPA axis and corticotropin-releasing factor [18].

Cocaine-induced changes and adaptations in the brain through repeated use will depend on the genetic background of each individual. Also, these functional modifications are modulated by environmental factors and the interplay between them and genetic risk factors. Thus, some individuals are more genetically susceptible to develop CUD and addiction than others, being only around 16–20% of cocaine users. In this review, we focus on the genetics of CUD.

## Genetic predisposition to cocaine dependence

### Heritability: family and twin studies

SUD, in contrast to other psychiatric disorders, are often seen as preventable and attributed to individual’s choice, but many studies have shown that they are heritable, highlighting the relevance of genetic risk factors [19]. Familial aggregation of alcoholism and addiction to illicit drugs is well described, and relatives of probands with SUD have an eightfold increased risk of drug use disorders, being 4.4-fold in the case of cocaine [20]. Familiar, adoption, and especially twin studies have brought consistent evidence that both environmental and genetic risk factors contribute to initiating the use of drugs of abuse, the transition to abuse, and the development of dependence. Heritability estimates described by different twin studies have shown that the genetic contribution to SUD and addictions is in general high, and also variable depending on the drug of abuse [21].

Heritability estimates for CUD are summarized in Table 2. In general, heritability is lower for drug use than for dependence for all drugs of abuse [19]. In the case of cocaine, the contribution of genetic factors for cocaine use is estimated ~0.39–0.44 [22–25], although higher estimates, 0.61–0.7, were found in other studies [26, 27] (Table 2). For cocaine abuse, heritability estimates are also highly variable, ranging from 0.32 to 0.79 [22, 24, 27–29]. Finally, genetic risk factors explaining the variance for cocaine dependence have been estimated to be consistently high across studies 0.65–0.79 [24, 27, 30] (Table 2), being one of the most heritable psychiatric disorders. Some of these studies estimated the contribution of common genetic risk factors for several substances of abuse, and the ones that are specific to cocaine, reporting a high percentage of 92–93% of common factors with other drugs [28, 29].

**Table 2:** Heritability estimates for cocaine use, abuse and addiction from twin studies.

Twin registry	Individuals	Phenotype	Prevalence	Heritability *h*^2^	Additive genetic factors a^2^	Shared environmental factors c^2^	Unique environmental factors e^2^	Ref.
Minnesota	94 MZ 94 DZ	CA/CD	1.5–12% CA/CD		0.39 CU males 0.74 CA/CD males 0.42 CA/CD females	0.18 CA/CD males 0.12 CA/CD females	0.08 CA/CD males 0.46 CA/CD females	[22]
Norwegian Institute of Public Health Twin Panel (NIPHTP)	668 MZ 718 DZ	CU	1.8%		0.44	0.33	0.23	[23]
Swedish nationwide registries and healthcare data	1720 MZ 1229 DZ 76,457 sibling pairs	Stimulant abuse (including cocaine)	1.7%		0.64 92% common^a^ 8% specific^a^	0.2 100% common^a^	0.16 60% common ^a^ 40% specific	[28]
Vietnam Era Twin (VET) Registry	1,874 MZ 1,498 DZ	CU and symptoms of CA	14.8% CU	0.43 felt dependent				[25]
Virginia Twin Registry	485 MZ 335 DZ (all females)	CU, CA, and symptoms of CD	14% CU 3.5% CA 2.4% CD		0.39 CU 0.79 CA 0.65 symptoms of CD	0.35 CU 0 CA 0 symptoms of CD	0.26 CU 0.21 CA 0.35 symptoms of CD	[24]
Virginia Twin Registry	708 MZ 409 DZ (all males)	CU, CHU, CA and CD	17% CU 3.5% CUH 5% CA 3% CD		0.61 CU 0.55 CHU 0.32 CA 0.79 CD	0.09 CU 0.23 CHU 0.32 CA 0 CD	0.3 CU 0.22 CHU 0.37 CA 0.21 CD	[27]
Virginia Twin Registry	1408 MZ 984 DZ	CU, CA, CD	17.4% CU 5.3% CA/ CD		0.56 CU (7% specific^a^) 0.63 CA/CD	0.14 CU 0 CA/CD	0.1 CU 0 CA/CD	[29]
Virginia Twin Registry	4826 twins	CU	9–29%		0.7	–	0.3	[26]
Virginia Twin Registry	2,817 MZ 2,048 DZ	symptoms of CA and CD	3–4% CA/CD	0.7 2% specific^a^				[30]

*MZ* monozygotic twin pairs, *DZ* dizygotic twin pairs, *CU* cocaine use, *CHU* cocaine heavy use, *CA* cocaine abuse, *CD* cocaine dependence

^a^Common/specific refers to factors shared with all drugs of abuse assessed in the study or specific to cocaine/stimulants.

### Genetic architecture of cocaine addiction

Although the contribution of genetic risk factors to cocaine addiction is very high, as described before, identifying the specific factors that underlie addiction is challenging due to the high polygenicity and complexity of this disorder. The contribution of common variants through association studies, as well as rare variants, has been investigated for cocaine dependence. Some relevant variants and genes have been highlighted, but most genetic risk factors are still largely unknown.

#### Common variants

Candidate gene association studies (CGAS) have focused on specific sets of genes based on a priori assumptions about their role in the mechanism of action of cocaine and in the development of addiction, including dopamine and serotonin neurotransmission (Supplementary Table S1). Although transgenic mice for some of these genes showed alterations in cocaine-seeking behavior, particularly those encoding the monoamine transporters [31], no robust associations have been found in CGAS. These association studies are typically underpowered and the significant associations identified so far have not been successfully replicated, except for the SNPs rs806368 in the *CNR1* gene [32–34] and rs16969968 in the *CHRNA5-CHRNA3-CHRNB4* gene cluster [35–37], found significantly associated with cocaine dependence in several studies with limited sample sizes (Supplementary Table S1).

So far, only one copy number variant (CNV) has been found associated with cocaine dependence, a large polymorphic CNV that partially spans the *NSF* gene, involved in synaptic vesicle turnover [38]. In this study, individuals with a low number of copies showed a quicker transition from cocaine use to dependence than individuals with a high number of copies.

The first genome-wide association study (GWAS) on cocaine dependence with positive findings was published in 2014 [39]. The authors developed a model (Sympcount_adj_) to remove the effect attributable to other substances (alcohol, opioids and nicotine), thus facilitating the identification of genetic risk variants for cocaine addiction. In addition, they used cocaine-exposed controls that did not develop addiction. In the European–American (EA) discovery sample (1809 cases and 292 controls), a genome-wide significant (GWS) association was found between cocaine dependence and rs150954431 (Table 3), located in the *NCOR2* gene (nuclear receptor corepressor 2) that regulates gene expression by activating histone deacetylase 3, and has been involved in memory [40]. However, this finding could not be replicated in two follow-up samples (including 4063 and 2549 subjects, respectively). The metanalysis of AA–EA samples revealed a different GWS hit on the *FAM53B* gene (family with sequence similarity 53 member B), rs2629540 (Table 3), that was almost GWS in the discovery sample. Although genetic variants in this gene have been related to changes in brain volumes [41], very little is known about *FAM53B* function and, more importantly, about its contribution to cocaine addiction susceptibility. Nevertheless, this association could not be replicated in a Spanish cohort of cocaine dependence (1011 cases and 1719 controls) [42]. Recently, a transcriptome-wide association study (TWAS) was performed using these data in several brain regions, although no significant associations were found in any tissue for cocaine dependence [43].

**Table 3:** Top findings on GWAS performed on cocaine use disorders.

rs ID	*P* value	Gene	Childhood environmental factors	Association	Sample	SNP heritability	Ref.
rs2629540	4.28E−08*	*FAM53B*	–	Dependence (DSM-IV)	2668 AA, 2101 EA	0.28	[39]
rs150954431	1.19E−09	*NCOR2*					
rs3075660	3.83E−07	*NRG3*	–	Dependence (DSM-IV)	6378 EA	0.27–0.30	[44]
rs10188036	1.77E−08	*TRAK2*	Change in residence	Dependence (DSM-5)	2998 AA	–	[6]
del-13:61274071	4.94E−08	*LINC00378*	Household drinking and illicit drug use				
del-1:15511771	3.61E−10	*TMEM51*	Nontraditional parental care	Subtype 5^a^	1916		
rs149843442	3.92E−08	*LPHN2*	Household tobacco use				
rs114492924	1.23E-08	*LINC01411*	Change in residence				
rs139389287	3.51E−08	*RP13–20L14.1*	Household tobacco use				
rs148834561	4.71E−08	*SLC7A13*	Household drinking and illicit drug use				
del-17:80342628	1.54E−08	*TRDN*	Change in residence				
rs75591854	3.91E−08	*SOGA2*	Household tobacco use/Change in residence				
rs75414569	9.73E−09	*RN7SL609P*	Household tobacco use				
rs148009780	4.74E−08	*SYNGR1*	Change in residence				
rs71428385	3.99E−08	*FN1*	Household tobacco use	Subtype 4^a^	3258		
rs56337958	3.07E−08	*TENM3*	Traumatic experience				
rs61835088 rs2825295	3.79E−09 2.57E−08	*FAM78B* *AL157359.3-AP000431.1*	–	Time to dependence (DSM-IV)	11,026 AA+EA 5020 AA	–	[47]

*EA* European–Americans, *AA* African–Americans.

*P value results from the combination of other replica samples used in the study.

^a^Subtypes 4 and 5 of cocaine use disorders resulting from cluster analysis.

A GWAS metanalysis of four samples of subjects with European ancestry (2085 cocaine-dependent patients and 4293 controls) found suggestive associations (*P* < 1E−05) of several SNPs with cocaine dependence, being rs3075660 in the *NRG3* gene the most significant one (Table 3) [44]. This gene, encoding a signaling protein involved in neurodevelopmental processes, had previously been associated with several psychiatric conditions, including schizophrenia (SCZ) [45]. In addition, at gene level, *HIST1H2BD*, encoding a histone protein, was found significantly associated with cocaine dependence [44]. Interestingly, this study estimated a SNP-based heritability (h^2^_SNP_) for cocaine dependence around 0.27–0.30 using two different approaches, and very similar results were obtained using GWAS data from Gelernter et al. (*h*^2^_SNP_ = 0.28; Table 3) [46].

Two GWAS on CUD have been recently published, one accounting for gene–environment interactions [6], and another one evaluating time to develop dependence [47] (Table 3). In the first one, two GWS hits were found associated with DSM-5 diagnostic criterion counts in AA individuals (*N* = 2998) [6], only when the interactive effect of childhood environmental risk factors were considered: change in residence for rs10188036 in *TRAK2* and household drinking and illicit drug use for del-13:61274071 in *LINC00378* (Table 3). In addition, a cluster analysis was performed to divide the sample in five CUD groups. For the subtypes 4 (*N* = 3258) and 5 (*N* = 1916; the highest heritable and heavy-cocaine-use clusters) they identified 11 additional GWS *loci* for which the effect on CUD was moderated by environmental factors (Table 3), highlighting the importance of considering environmental interactions in the statistical models [6]. In addition, this study supports the idea that CUD subjects can be decomposed into different subgroups, as previously mentioned, and the study of more homogeneous subgroups may result in more powerful genetic analyses [5]. The second one identified two GWS hits associated with time from cocaine use to dependence onset, rs61835088 identified in the meta-analysis of EA–AA individuals, and located in the gene *FAM78B*, and rs2825295 in AA individuals [47]. This approach highlights the importance of investigating addiction-related phenotypes to uncover genetic risk factors involved in the development of dependence.

Today, the most important limitation for the association studies on cocaine addiction is the sample size. Hypothesis-free association studies, in the form of GWAS, have taken over from the old CGAS, allowing identification of new, unexpected associations that would have never been explored in hypothesis-driven studies. Nonetheless, a GWAS involves testing millions of genetic variants, which imposes a highly astringent statistical price. This, together with the fact that psychiatric disorders are highly polygenic, with each variant contributing individually to a small amount of the risk (odds ratios typically < 1.3), makes it necessary to gather tens of thousands of patients and controls to achieve appropriate statistical power [48]. Compared to other substances misuse, the sample sizes of GWASs of CUD are substantially smaller. For example, GWAS on problematic alcohol use (about 435 K participants) [49], opioid use disorder (~80 K) [50], cannabis lifetime use (~180 K) [51], and several stages of tobacco and alcohol use (~1.2 M individuals) [52], have been performed so far. When we look at other psychiatric conditions, we find that the most recent GWAS on attention-deficit/hyperactivity disorder (ADHD, 20 K cases) and autism spectrum disorders (ASD, 19 K cases) yielded only 12 and 5 independent GWS hits, respectively [53, 54]. Currently available GWAS for cocaine phenotypes are all below 11 K samples, and therefore larger sample sizes are needed for robust findings.

Due to the difficulty in recruiting patients that are dependent only on cocaine, some authors have studied the general genetic liability of several illicit drugs of abuse (e.g., cocaine, cannabis, and opioids) by combining individuals addicted to any of them [55–59]. These studies identified several SNPs that show a significant association with the phenotype, with subsequent replication in independent samples. However, the sample size of these studies is still limited and further studies are needed.

Another important and controversial consideration in association studies for SUD is the selection of the control sample. Some studies use control individuals that have been exposed to the drug at least once in their life [39], hence excluding the genetic risk factors related to impulsivity or risk-taking behavior, which are very important in this disorder, as they facilitate the first contact with the drug. Indeed, genetic correlation analyses have recently shown the existence of shared genetic factors between cocaine addiction and risk-taking behavior [44]. For that reason, other studies used screened controls that do not meet the DSM criteria for addiction or unselected controls from the general population [44, 57, 60]. The last approach could eventually dilute positive findings due to the presence of some cases among the controls; however, based on the prevalence of cocaine dependence in the general population (about 1.1%), the occurrence of false-negative results due to this effect is likely to be neglectable.

As previously mentioned, the prevalence of CUD varies among ethnicities. Two of the three GWAS performed on CUD [6, 39] included both AA and EA individuals, and their results, as well as those from other SUD [57], suggest that some genetic risk variants can be associated with the phenotype only in a particular population. Although this could be due to limited sample size, further studies in larger samples and considering different ethnicities are warranted.

While GWAS have begun to identify genes that underlie several traits relevant to drug abuse, they still explain only a small fraction of the heritability of this disorder. One of the most important limitations of these studies is the intrinsic difficulty in obtaining large and homogeneous human samples. This has fostered the emergence of a new project (www.ratgenes.org) that will perform GWAS on different behavioral traits relevant to drug abuse using thousands of male and female outbred rats (heterogeneous stock rats). A similar approach has already been taken on obesity‐relevant traits [61]. In addition, expression data of several brain regions relevant to the addiction process will be analyzed to identify expression QTLs (eQTLs). This approach can help to identify new genes that influence drug abuse-related behaviors in rats, providing potential candidates for the genetic susceptibility to drug addiction in humans.

#### Rare variants

Only two studies have explored the contribution of rare (Minor Allele Frequency, MAF<1%) and low-frequency variants (1%<MAF<5%) to cocaine addiction. The first one, with focus on the family of acetylcholine receptors, found increased DSM-IV cocaine dependence symptoms among carriers of rare missense variants in *CHRNB3*, coding for the β3 nicotinic acetylcholine receptor [62]. The other one, focusing on the gene encoding the μ-opioid receptor (*OPRM1*), revealed an association between rs62638690 and cocaine addiction [63]. Further studies with larger samples sizes that use methodological approaches other than GWAS are needed to dissect the role of rare genetic risk variants on cocaine dependence.

### Shared genetics of CUD with other psychiatric conditions and traits

Recognition of co-occurring psychiatric disorders among people with SUD has been growing in recent years. Several studies have shown that SUD is highly comorbid with other psychiatric disorders such as SCZ, major depressive disorder (MDD), or ADHD [64–67], and traits like aggressive, antisocial, or risk-taking behaviors [68, 69]. For example, about 81% of SUD patients have at least one comorbid mental disorder: 33% MDD, 11% SCZ, and 9% personality disorders [64]. Conversely, the occurrence of lifetime SUD in patients with SCZ is 70–80%, 39.2% for ADHD, and 16.1% for MDD [65–67]. The pattern is similar with cocaine: 73.4% of cocaine abuse/dependence patients have comorbid mental disorders (49.7% personality disorders, 23.4% MDD, and 20.5% anxiety), and 94.9% have other SUD [70]. Such comorbidities determine a worse lifetime trajectory in patients, lower rates of treatment success, and a higher prevalence of suicide. Several studies have started to investigate whether shared genetics is behind these comorbid patterns using different statistical tools, such as the estimation of genetic correlation between phenotypes, polygenic risk score (PRS) analysis to quantify the fraction of the genetics of a given phenotype that predicts a second condition, and Mendelian Randomization or Latent Causal Variable model to infer causal relationships. These analyses support the genetic overlap between different SUD and other mental disorders, including SCZ, ADHD, MDD, bipolar disorder (BD), eating disorders, or insomnia, among others [71–80]. However, studies focusing particularly on cocaine are still scarce due to the limited availability of properly sized GWAS.

A recent GWAS metanalysis on cocaine dependence [44] explored genetic correlations between this phenotype and six previously described comorbid conditions and found significant results for SCZ (rg = 0.2 ± 0.05), ADHD (0.5 ± 0.08), MDD (0.4 ± 0.08), and risk taking (0.35 ± 0.06). Testing 690 traits from LDHub [44] yielded 109 significant findings, including negative correlations with cognitive phenotypes (e.g., college completion) or reproductive traits (e.g., age at first child) and positive correlations with several psychological or psychiatric phenotypes like neuroticism, depressive symptoms, or loneliness. Also, PRS for SCZ, ADHD, antisocial behavior, MDD, risk-taking and children’s aggressive behavior were found associated with cocaine dependence (pseudo-*R*^2^ = 2.28%, 1.39%, 1.33%, 1.21%, 0.60%, and 0.30%, respectively) [44]. Several other PRS studies with focus on SUD included also analyses of a cocaine sub-sample: PRS for SCZ (but not for BIP) associated with CUD in a study of Icelandic subjects (R^2^ = 0.62%) [75], and PRS for SCZ associated with stimulant use disorders in subjects from the Family Study of Cocaine Dependence (FSCD) (pseudo-*R*^2^ = 1.7–3.5%) [73]. Finally, PRS for five psychiatric disorders (SCZ, ASD, ADHD, MDD, and BD) were tested in different cocaine phenotypes from the FSCD, the Collaborative Study of the Genetics of Nicotine Dependence (COGEND), and the Collaborative Study of the Genetics of Alcohol Dependence (COGA) samples. Most associations found were driven by general substance liability, but some substance-specific associations were also identified between MDD, BD, and SCZ PRSs and severe cocaine dependence [71].

Pleiotropy has also been reported at the level of single genes: For example, a missense polymorphic variant (rs16969968:G>A, p.Asp398Asn) at *CHRNA5*, encoding the α5 nicotinic acetylcholine receptor, leads to hypofunction of the protein and is likely the most robust and replicated association with risk for nicotine addiction [81]. This SNP has also been found associated with cocaine addiction in several studies [35–37], but the direction of the effect is opposite, with allele A being a risk factor for nicotine addiction but protective for cocaine addiction [35]. The molecular basis of this distinction is not clear but seems to be related with the localization of this receptor in the mesolimbic dopamine system (both in excitatory dopaminergic and in inhibitory GABAergic neurons) and the mechanism of action of nicotine and cocaine [35]. Interestingly, antagonists of the receptor decrease the reinforcing effects of cocaine, whereas *Chrna5* knock-out mice have an increased intake of nicotine [82, 83].

To summarize, we know that CUD is highly comorbid with other psychiatric disorders and cognitive or personality traits, but the role of shared genetics on these co-occurrences has been poorly studied. Thus, we do not know whether risk alleles are acting independently on CUD and on its comorbid phenotypes (i.e., horizontal pleiotropy, also known as biological pleiotropy) or whether one phenotype is causally related to the other one, so that the variants associated with one condition are indirectly associated with the second one (i.e., vertical pleiotropy, also known as mediated pleiotropy). The latter may be exemplified by genetic risk factors contributing to ADHD, which in turn would lead to the onset of CUD under the hypothesis that subjects with ADHD use cocaine to reduce symptomatology (“self-medication” model of comorbidity) [84]. Using analytical tools that investigate causal relationships is needed to clarify this issue.

Another relevant issue is whether the different SUD share genetic risk factors or if substance-specific factors are predominant. In this regard, the fact that heritability estimates for substance-related phenotypes significantly differ depending on the drug of abuse supports the view that at least certain risk factors may be substance-specific [85]. However, there is evidence from twin studies that a large proportion of genetic liability is shared across substances [29, 86], and molecular studies show that increased cross-disorder polygenic risk (e.g. from psychopathological conditions) would be associated with greater general substance involvement [71].

## Changes in gene expression induced by cocaine

Cocaine use induces changes in the structure and function of the brain, such as neuronal connectivity and synaptic plasticity [87–90]. Some of these changes may become stable, contributing to addiction and relapse in cocaine use. Epigenetic and gene expression changes underlie these neuroadaptations induced by the drug and help to explain functional alterations [91, 92].

### Transcriptomic studies

Several studies have assessed gene expression alterations in *postmortem* brain samples from cocaine abusers or in neuronal cells in vitro, the vast majority using microarrays (Table 4). It should be mentioned that these studies are based on few individuals due to the inherent difficulty in obtaining these samples. Across the different studies, some functions are recurrently identified among the genes that are differentially expressed, such as transcription regulation [93–99] and signal transduction [93–96, 99–101], but certain functions and pathways have been identified only in some particular studies.

**Table 4:** Transcriptomic alterations induced by cocaine in *postmortem* brain samples of cocaine abusers or cell lines exposed to cocaine in vitro.

Brain region/ cell type	Samples	Method	Number of altered transcripts	Multiple testing correction	Molecular functions and pathways	Validated Genes (qPCR)	Ref.
Anterior prefrontal cortex (aPFC)	42 cocaine, cannabis, and/or phenycyclidine abuse 30 controls	Mammalian Gene Collection (MGC) arrays	160 (shared by all three)	None	Calmodulin signaling, Golgi and endoplasmatic reticulum-related, lipid metabolism	*CALM2* *APOL2* *SEMA3B*	[100]
Dorsolateral prefrontal cortex (dlPFC)	7 cocaine abusers 7 controls	Neuroarray	65	None	Cytoskeleton and extracellular matrix, mitochondrial function and energy metabolism, glycoproteins and oligodendrocyte-related, neurotransmission and signaling, transcription factors, drug metabolism, phosphatases and kinases	*GNAO1* *MDH1* *MAP1B* *PLP1*	[93]
Nucleus accumbens (NAc)	10 cocaine users (5 abusers) 10 controls	Affymetrix oligonucleotide arrays: u95Av2, u95B, u95C, u133A and u133B	49	None	Signal transduction, transcription translation and RNA processing, Neurotransmission, synaptic function, membrane recycling, glial, cell adhesion, receptors, transporters, channels, immune and stress response, protein processing and degradation and lipid-related	*CART* *MBP* *MOBP* *PLP1*	[94, 95]
Hippocampus	10 cocaine abusers 11 controls	Affymetrix oligonucleotide arrays: u133A and u133B	242	5% FDR	Cell adhesion, extracellular matrix, receptors and signal transduction, neurogenesis and axon guidance, angiogenesis, apoptosis and cell death, transcription and translation, ion channels, and transport	*RECK* *PCDH8* *CTGF* *EPHB4* *SGKL* *HCN2* *LAMB1* *OPHN1* *ITGB6* *CTNNBIP1*	[96]
Hippocampus	9 chronic cocaine addicts 8 alcoholics 9 controls	RNA-seq	394 (cocaine) 29 (shared alcohol)	20% FDR	Mitochondrial inner membrane function, oxidative phosphorylation, negative regulation of gene expression, gene silencing, RNA-binding, covalent chromatin modification, extracellular matrix part, long term potentiation	*HIST1H4E* *CDR1* *BEX4* *CACNB2* *NDUFA5* *NDUFAF1* *SNX27* *GRIN2B* *HNRPH1* *CAMK2D* *DDX17* *MDM4*	[97]
DA cell-enriched regions (substantia nigra, ventral tegmental area)	10 cocaine abusers 10 controls	Illumina array HT-12 BeadChips	91	5% FDR	Chromatin organization, behavior, drug response, transcriptional regulation, negative regulation of cell death, unfolded protein response and RNA catabolism, DA metabolic processes, and neuronal differentiation	*CDKNIA* *CCL2* *JUN* *GADD45B* *GSTMI* *PVALB* *SLC6A3* *KCNJ6* *MRAP2* *TH* *FOXA2*	[98]
Neuronal progenitor cells	HPNC - Human neuronal progenitor cells 30′ exposure to: 1 μM cocaine 5 µM cocaine Evaluated at 6 h, 24 h and 48 h	Affymetrix oligonucleotide arrays: u133A	5 µM: 1344 (6 h) 1073 (24 h) 0 (48 h) 1 μM: 2449 (6 h) 736 (24 h) 51 (48 h)	5% FDR	6 h after acute exposure: cell maintenance, cell cycle, DNA replication, recombination and repair, cell growth and proliferation. Cell death and cancer only at higher dose 24 h after acute exposure: Immune response, Inflammatory disease, immunological disease, infection disease, hematological disease, cancer and cell death, viral function and infection, ERK/MAPK signaling	*CXCL2* *ICAM1* *IFIT1* *IL8* *MX1* *NFkB1* *NFkB2* *OAS1* *STAT1* *STAT2* *TNFa*	[101]
Dopaminergic neuronal cells	SH-SY5Y differentiated to dopaminergic 30′ exposure to: 1 μM cocaine 5 µM cocaine Evaluated at 6 h and 24 h	Affymetrix GeneChip Human Genome U133 Plus 2.0 Array	5 µM: 756 (6 h) 0 (24 h) 1 μM: 0 (6 h) 0 (24 h)	10% FDR	Regulation of transcription, chromatin modification and organization, focal adhesion, adherens junction, cell projection, neurotrophin signaling, MAPK signaling, cellular response to stress, proteolysis, cell cycle, intracellular and protein transport	*ENC1* *NFAT5* *ELF1* *PPP1R9A* *IGF2BP3* *NRG1* *SEMA6D* *miR-124-3p* *miR-124-5p* *miR-137 miR-101-3pmiR-9-5p miR-369-3p miR-153-3p*	[99] [102]

*FDR* false discovery rate.

Alteration of gene expression in the prefrontal cortex of cocaine abusers was assessed in two studies, using *postmortem* samples of dorsolateral and anterior prefrontal cortex (dlPFC and aPFC, respectively). The first study focused on expression alterations in aPFC shared in cocaine, cannabis, and phencyclidine abuse, highlighting genes related to calmodulin signaling, Golgi and endoplasmic reticulum [100]. In dlPFC, expression changes identified in cocaine abusers involved genes implicated in mitochondrial and oligodendrocyte function, among others [93]. Interestingly, in the NAc of cocaine abusers, myelin-related genes and genes involved in glial function were also found altered, consistent with a loss of MBP-positive oligodendrocytes [94], and alterations in the expression of *PLP1*, encoding a major constituent of myelin, were identified [93–95].

Furthermore, expression changes in neurotransmission-related genes were identified in dlPFC and NAc [93–95], in particular genes involved in synaptic function or cell adhesion [94, 95]. In hippocampus of cocaine abusers, two different studies detected alterations in the expression of genes involved in the regulation of the extracellular matrix [96, 97], and also in cell adhesion, neurogenesis and axon guidance [96], or mitochondrial function, oxidative phosphorylation and long-term potentiation [97]. Another study using *postmortem* brain samples assessed the expression in midbrain of dopamine cell-enriched regions identifying alterations in expression related to dopamine metabolic process and neuronal differentiation [98] (Table 4). Remarkably, the first study also assessed the substance-specific and shared gene expression changes between cocaine and alcohol in hippocampus, observing that cocaine induced many more transcriptomic alterations than alcohol, but also identifying common changes in the same direction for both drugs that were related to neuroadaptations [97].

The effects of cocaine on human gene expression have also been evaluated in human cells in vitro. Human neuronal progenitor cells showed alterations in the expression of genes mainly involved in immune and inflammatory processes and cell death when exposed to cocaine [101], in line with several studies mentioned above [94–96, 98]. In a dopaminergic neuron-like model, changes in gene expression involved chromatin modification [99], also occurring in dopamine cell-enriched regions and hippocampus [97, 98]. Other functions were also identified such as cell cycle, adhesion, cell projection and neuroadaptations [99], and epigenetic regulation by several miRNAs could explain, at least partially, the expression changes induced by cocaine [102] (Table 4).

Gene expression changes induced by cocaine have been widely investigated in animal models, adding valuable information to human studies and contributing to the understanding of the molecular changes involved in the development of CUD. Convergent gene expression changes between animal and human studies have highlighted pathways and biological processes that may be relevant for CUD, including the ERK/MAPK signaling pathway, long-term potentiation, synaptic plasticity (synucleins), and mitochondrial function [103]. Other studies have used the data generated by the above-mentioned studies in humans to assess convergence of gene expression mechanisms with rodents, highlighting genes involved in dopamine and serotonin function such as *SLC1A2, CALM3, ALDOA, ALDOC*, and *ENO2* [104] and in brain plasticity like *APP, GRIN2A, GRIN2B, KCNA2, MAP4, PCDH10, PPP3CA, SNCB*, and *SV2C* [105].

It should be mentioned that several of the studies previously performed in humans did not apply multiple testing corrections, a relevant statistical filter when thousands of transcripts are interrogated. Nevertheless, all of them validated expression differences of selected genes by quantitative real-time PCR (Table 4). It is important to note that biological samples from cocaine-addict individuals are difficult to obtain and involve a wide range of variables that cannot be controlled and add heterogeneity, such as dose and frequency of use, time of last exposure, use of other drugs or cause of death. In contrast, in vitro experiments evaluating cocaine effect on cell lines allow to control for variables such as cocaine concentration and time of exposure, but they cannot mimic the effect of chronic cocaine use in the brain of an addicted individual and the effect of the crosstalk with other cell types and remodeling of circuits. By the use of novel techniques, such as single-cell RNA-sequencing, *postmortem* brain regions could be used to dissect which cell types show more relevant expression changes in each area and, then, study in vitro the effect of cocaine in a controlled environment using specific iPSCs-derived models. Moreover, the information that can be obtained from a few human samples is limited, making it difficult to fully understand the changes in gene expression and in the biological processes that occur in the brain of cocaine users. Larger biorepositories are needed that gather *postmortem* brain samples for CUD (and other drugs of abuse) with properly phenotyped individuals, similar to other initiatives, such as PsychEncode or GTEx (http://resource.psychencode.org/ and https://gtexportal.org, respectively). Due to the intrinsic difficulties in obtaining expression data from the brain of patients, several tools have been developed to use transcriptomic imputation to integrate genotype and expression data from large consortia, like GTEx, through machine learning. These TWAS approaches allow identification of regulatory variants associated with a given disorder, such as CUD.

### Epigenetics

As mentioned above, cocaine addiction is a maladaptive neural plasticity process that occurs in response to repeated drug exposure in vulnerable individuals, depending on the genetic and environmental risk factors, and their interaction. Epigenetics is a vehicle through which environment, including the effects of drugs of abuse, interacts with an individual’s genome to reversibly regulate gene expression independently of the DNA sequence, and determine aspects of function, in health and disease, including addiction. Several studies have demonstrated cocaine-induced changes in epigenetic mechanisms like histone modifications, DNA methylation, and microRNAs, all of them recently reviewed [92, 106–111]. However, only a few studies have been conducted in human samples. Regarding histone posttranslational modifications, we found only one study that inspected genome-wide changes in H3K4Me3 in *postmortem* hippocampus of individuals with chronic exposure to cocaine, revealing changes in promoters of 1115 genes, although only five of them overcame multiple testing corrections [97]. On the other hand, a recent study examined DNA methylation profiles in the peripheral blood of CUD patients and found 186 differentially methylated positions, proposing these regions as potential biomarkers [112]. Finally, two studies investigated changes in the expression of microRNAs induced by cocaine in human cultured cells [102, 113] and two others in peripheral blood and in *postmortem* brains of cocaine abusers [114, 115]. Interestingly, all of them found alterations in the expression of miR-124, a miRNA that has also been related to cocaine effects in rodents [116, 117]. Further studies are needed to explore the epigenetic mechanisms that underlie cocaine addiction and identify potential biomarkers and therapeutic targets.

## Convergence of expression and genetic studies

Genes which expression is altered by cocaine, and that possibly mediate its effects and neuroadaptations induced in the brain, could hold genetic risk variants that contribute to the susceptibility to cocaine addiction. These risk variants may have an impact on the expression or function of these genes prior to the use of the drug and/or confer a differential response to cocaine that could be relevant for the establishment of changes in neuronal circuits, necessary for the development of addiction. Under this hypothesis, some recent studies have pinpointed genes that show altered expression and bear genetic risk variants.

Expression of *NFAT5* was found increased in dopaminergic neuron-like cells upon acute exposure to cocaine and, additionally, five SNPs in this gene were associated with cocaine dependence [99]. NFAT5 (TonEBP) is a transcription factor, and previous evidence suggests that cocaine-induced activation of gene expression may be mediated, in part, by NFAT-dependent transcription [118].

Three miRNAs, miR-9, miR-153, and miR-124, were downregulated by cocaine in the above-mentioned dopaminergic model, possibly regulating expression changes observed in the previous study. Interestingly, these miRNAs were found associated with cocaine dependence in a gene-based association study [102].

*PLCB1*, Phospholipase C beta 1 protein, carry genetic variants associated with both cocaine dependence [119] and drug dependence [119, 120]. Increased expression of *PLCB1* is found in both the NAc of human cocaine abusers and in cultured dopaminergic-like human neurons treated with cocaine [119]. Increased expression has been also found in the NAc of mice self-administering cocaine and during withdrawal [121], and a recent study in mice suggests that this gene may play an important role in relapse to cocaine consumption [122].

*KCTD20*, a regulator of AKT signaling, was identified in a recent study that combined genomic and transcriptomic data to detect candidates for cocaine addiction. This gene was one of the three GWS findings in a GWAS of cocaine dependence [39], and altered expression was found in hippocampus of cocaine abusers, being a key node in a gene network associated with human cocaine use [46]. This gene is a member of the KCTD family, which is involved in a wide range of processes, including proteasome function, GABA signaling, and regulation of transcription responses.

These studies seem to support the hypothesis that genes mediating cocaine’s effect can also participate in the vulnerability to addiction. But the number of works is still limited and further studies are needed to investigate the convergence between expression and genetic studies. Research in the field would benefit from large GWAS and transcriptomic studies and the use of integrative analyses (e.g., TWAS) to get more insight on the genetic basis of this disorder.

## Treatment and pharmacogenetics

Currently, there is no government-approved medication available to treat CUD. The medications tested in clinical trials target multiple systems altered by cocaine, including (1) dopamine agonists, as releasers or uptake inhibitors; (2) antagonists/blockers, as antipsychotics, cocaine vaccine, GABA modulators, noradrenergic agents (e.g., disulfiram, doxazosin); (3) other approaches (e.g., cholinesterase inhibitor and N‐methyl‐D‐aspartate receptor antagonist); and (4) combination of medications (reviewed by [123, 124]). However, the efficacy of these treatments requires further exploration [125].

Pharmacogenetic approaches investigate the genetic factors responsible for the inter-individual medication response variability. The final aim of pharmacogenetics is to identify the most effective “personal therapy” based on the genetic individual background, minimizing medical side effects, ensuring compliance, and adjusting dosage [86, 126]. This core element of personalized medicine is a reality in the oncology field. Patient’s specific cancer driver genes can be used as biomarkers to select the best-suited therapy in terms of response to treatment and prognosis [127]. The identification of these correlations has been possible thanks to huge consortiums, such as the Pan-Cancer Atlas, studying thousands of samples in depth [128].

The influence of genetic variants in treatment response in SUD is still at an early stage. An illustrative example is the study of the interaction genotype-treatment in smokers [129]. In African Americans, smokers with the GG genotype at the missense SNP variant rs16969968 in *CHRNA5* responded better to a combination nicotine replacement therapy, whereas for A carriers the efficacy of varenicline was higher [130]. Although these findings require additional replications, selection of medication based on genotype could lead to higher rates of smoking cessation.

Early CUD pharmacogenetic studies focused on mouse models (reviewed by [131]). Now, some double-blind randomized placebo-control trials in humans have been performed to analyze cocaine drug treatment response based on the genotypes of specific functional variants on ten genes (*ADRA1A*, *ADRA1D, ANKK1*, *DRD2*, *DBH*, *MTHFR*, *OPRK1*, *THP2, SLC6A4*, and *SLC6A3*) related with cocaine physiological mechanisms [132–143]. The medications assessed include doxazosin, an α1A adrenergic antagonist that decreases noradrenergic activity; levodopa, a dopamine precursor; cocaine vaccine, succinylnorcocaine covalently linked to cholera B [137]; and disulfiram, a cooper chelator that inhibits noradrenergic synthesis. The results of these studies are summarized in Table 5. Extensive reviews of cocaine medication pharmacogenetics have been published [123, 144–146]. However, the knowledge in the field is still in its early stages.

**Table 5:** Studies testing the relationship between genetic variants and pharmacotherapies for cocaine substance abuse treatment (adapted from Patriquin et al. [146]).

Gene	Polymorphism	Variant (nt and position)	Allele: functional effect	Study design: time, dose, sample (medication+placebo)	Genotypes: treatment; placebo	Substance/s abused	Pharmacotherapy/active immunotherapy	Results	Ref.
*ADRA1A*	rs1048101	T>C p.Cys347Arg	May alter receptor activity	12 weeks 250 mg/day *n* = 69(32 + 37)	7CC, 25CT/TT; 15CC, 22CT/TT	C–O	Disulfiram (stabilized on methadone) + CBT	CT/T: reduced cocaine-positive urines rates (84% to 56%) on disulfiram. CC no difference	[132]
*ADRA1D*	rs2236554	T>A 3′UTR	T: lower receptor density	12 weeks 8 mg/day *n* = 76 (47 + 29)	21AA, 26AT/TT; 19AA, 10AT/TT	C only	Doxazosin + CBT	AT/TT greater decrease cocaine-positive urines rates on treatment.	[133]
*ANKK1*	rs1800497# (Taq IA)	C>T p.Glu713Lys	T: more thermostable enzyme	*12 weeks 250 mg/day *n* = 68 (31 + 37)	18 CC, 13CT/TT; 17CC, 20CT/TT	C–O	Disulfiram (stabilized on methadone) + CBT	CT/TT: reduced cocaine-positive urine rates (80% to 52%) on disulfiram. CC no difference	[139]
*DRD2*	rs2283265#	G>T intronic	T: lower receptor density	*12 weeks 250 mg/day *n* = 68 (31 + 37)	23GG, 9GT/TT; 22GG, 14GT/TT	C–O	Disulfiram (stabilized on methadone) + CBT	GT/TT: reduced cocaine-positive urine samples (67 to 48%) on disulfiram. GG marginal decrease. *DRD2*&*ANKK1* one minor allele at least in one associated to better response	[139]
*DBH*	rs1611115	C>T −1021 5’UTR	T: lowest DBH levels circulating	*12 weeks 250 mg/day *n* = 75 (35 + 40)	17CC, 17CT/TT; 21CC, 19CT/TT	C–O	Disulfiram (stabilized on methadone) + CBT	CC reduced cocaine-positive urine rates. CT/TT no difference.	[136]
*DBH*	rs1611115	C>T −1021 5’UTR	T: lowest DBH levels circulating	12 weeks 5× 360 mg vaccines *n* = 71 (34+37)	18 CC, 16CT/TT; 19CC, 18CT/TT	C–O	Cocaine vaccine (succinylnorcocaine covalently linked to cholera B SNC-rCTB) + CBT	CT/TT: reduced cocaine-positive urine rates (77% to 51%) on vaccine. Decreased on CC (83% to 72%). Placebo no differences.	[137]
*DBH*	rs1611115	C>T -1021 5′UTR	T: lowest DBH levels circulating	12 weeks 8 mg/day *n* = 76 (47 + 29)	33CC, 14CT/TT; 16CC, 13CT/TT	C only	Doxazosin + CBT	CT/TT: reduced positive-cocaine urine rate with greater efficacy vs CC.	[134]
*DBH*	rs1611115	C>T -1021 5′UTR	T: lowest DBH levels circulating	12 weeks *n* = 71 (38 + 33)	26CT/TT, 12CC; 20CC, 13CT/TT	C only	Levodopa-carbidopa	CT/TT reduced cocaine-positive urine rate on levodopa/carbidopa pharmacotherapy. CC no effect. Placebo no effect.	[138]
*DBH*	rs1611115	C>T -1021 5′UTR	T: lowest DBH levels circulating	12 weeks 250 mg/day *n* = 177 (91 + 86)	40CC, 40CT/TT; 44CC, 29CT/TT	C–O	Disulfiram (buprenorphine maintenance treatment)	Less frequent cocaine use on disulfiram. No significant differences in cocaine-negative urine tests or consecutive weeks abstinence. Limited support for the efficacy of disulfiram for reducing cocaine	[135]
*MTHFR*	rs1801133	C>T 677	T: enzyme less active and higher homocysteine levels	*12 weeks 250 mg/day *n* = 67 (32 + 35)	14CT/TT, 10CC; 18CT/TT, 14CC	C–O	Disulfiram (stabilized on methadone) + CBT	CT/TT reduced cocaine-positive urines (79% to 52%) on disulfiram. CC genotypes also modest reduction.	[140]
*OPRK1*	rs6473797	A>G	Unknown	16 weeks 5× 360 mg vaccine *n* = 66 (33+33)	20AA, 13AG/GG; 18AA, 15AG/GG	C–O	Cocaine vaccine (succinylnorcocaine covalently linked to cholera B SNC-rCTB)	AA reduced cocaine-positive urines (78 to 51%) on using the vaccine. Vaccine treatment remained effective only in the AA group	[141]
*THP2*	rs4290270	A>T 1125	A: lowest serotonin synthesis	*12 weeks 250 mg/day *n* = 68 (31 + 37)	22AA/AT, 9TT; 22AA/AT, 15TT	C–O	Disulfiram (stabilized on methadone) + CBT	AA/AT reduced cocaine-positive urine rate (80% to 64%) on disulfiram. TT no differences. *SLC6A4*&*THP2*: S’ carriers & A reduced cocaine urine levels (71% to 53%) on disulfiram.	[142]
*SLC6A4*	5-HTTLPR (rs4795541/rs25531)	Short, Long A>G. S′ = LG + S, L′ = LA	S′: lower expression	*12 weeks 250 mg/day *n* = 71 (33 + 38)	27S′S′/L′S′, 6L′L′; 30SS′/L′S′, 8L′L′	C–O	Disulfiram (stabilized on methadone) + CBT	S’S’/L’S’ group reduced cocaine-positive urine rated (78% to 54%) on disulfiram. L’L’ no difference.	[142]
*SLC6A3*	rs28363170	VNTR 40 bp x9/10 repeats 3′UTR	9: greater expression	12 weeks 250 mg/day *n* = 67 (32 + 35)	15 (10–10), 17 (9–10/9–9); 17 (10–10), 18 (9–10/9–9)	C–O	Disulfiram (stabilized on methadone) + CBT	10/10 reduced cocaine-use (78% to 48%) and decreased cocaine-positive urine rates in disulfiram. No differences in placebo.	[143]

*Same sample; # variants in proximity (*R*^2^ ~0.57).

*C* cocaine, *O* opioid dependence, *CBT* cognitive behavioral therapy, *VNTR* variable number tandem repeats. *ADRA1A* Adrenoreceptor Alpha 1A, *ADRA1D* Adrenoreceptor Alpha 1D, *ANKK1* Ankyrin repeat and kinase domain containing 1, *DRD2* Dopamine receptor D2, *DBH* Dopamine β-hydroxylase, *MTHFR* Methylene tetrahydrofolate reductase, *OPRK1* κ-Opioid receptor, *THP2* Tryptophan hydroxylase, *SLC6A4* Serotonin transporter, *SLC6A3* Dopamine transporter 1 (*DAT1*).

Interestingly, the SNP rs161115 (-1021C>T) in the dopamine β-hydroxylase gene (*DBH)*, responsible for up to 50% of plasma activity variation of the enzyme, has been analyzed in multiple studies. Different clinical responses for each allele (C/T) were observed when distinct treatments were used [134–138], suggesting that the individual’s genotype could help to make treatment choices. For instance, individuals with the CC genotype, associated with normal DBH levels, presented significant cocaine-positive urine reduction rates on disulfiram treatment in one study [136], although the results were not supported by another study [135]. Contrarily, T-allele carriers (CT and TT genotypes), associated with lower levels of circulating DBH, seem to respond better to doxazosin, levodopa, or cocaine vaccine medications [134, 137, 138].

The responses to treatments considering multiple polymorphic variants in different genes at the same time are of particular interest. These interactions were tested in two studies [139, 142]. The analysis of genotype combinations at *ANKK1* rs1800497 and *DRD2* rs2283265, in low linkage disequilibrium (*R*^2^ = 0.57), showed that carrying at least one minor allele in one of these SNPs is associated with better response to disulfiram [139]. In the other study, carriers of the S′ allele at the serotonin transporter (*SLC6A4*) 5-HTTLPR polymorphic variant and the A allele at the rs4290270 SNP of tryptophan hydroxylase (*THP2*), both corresponding to the low activity variants, presented significant reduction in cocaine urine levels (71–53%) on disulfiram treatment [142].

Pharmacogenetic studies published until now present some limitations. (1) The samples sizes are very limited, particularly when compared to GWAS. This is due both to the cost of the clinical trials, and to the limited adherence/abandonment of participants. (2) Multiple testing corrections are not systematically applied as each study focus on one or two functional variants. (3) Only for *DBH* more than one article has studied the gene/variant. Therefore, replication of the other results in independent samples is needed to confirm the participation of these polymorphisms in differential drug response. (4) The populations studied are ethnically diverse but the groups are not similarly represented. However, population stratification has been assessed and taken into consideration in statistical analyses. Larger samples representing the multiple ethnic groups are warranted to extract generalizable conclusions. (5) Incomplete knowledge on the effect of gene variants, and on the physiopathology of cocaine use dependence. (6) The drug may not be selective for a specific target, making the individual genetic variant approach insufficient. In addition, in some studies, multiple drugs were given (e.g., disulfiram and methadone). (7) Finally, individuals participating in the study present comorbidities with additional SUD (alcohol, opioids, etc.), although comorbid psychiatric disorders have been specifically excluded. Larger and more comprehensive studies are needed to be able to move toward personalized medicine in cocaine use disorder therapy.

## Concluding remarks

In the last decades, GWAS have been extensively used to study complex disorders such as cocaine dependence; however, common variants identified so far by this approach only explain a fraction of its genetic liability, probably around 30% [44, 46]. To identify the molecular components that underlie the “missing heritability” of cocaine dependence we need to (1) significantly increase the sample size of GWAS, (2) consider other types of genetic variation that cannot be properly addressed in those studies, including structural variants (e.g., CNVs) and rare variants that have been poorly investigated so far, (3) explore epistasis, i.e., interaction among genes, (4) investigate epigenetic mechanisms, as well as gene-environment interactions, (5) combine GWAS results with functional data such as expression data, 3D chromatin interaction and/or regulatory histone marks to identify most likely causal variants, (6) improve the classification of cases in different subgroups (e.g., using cluster analysis rather than DSM-5 criteria) to have more homogeneous groups and increase the statistical power of genetic studies and (7) use new reference imputation panels for GWAS, such as the Haplotype Reference Consortium [147] or TOPMed [148], that improve the quality of the imputation of common genetic variants and provide a better coverage of rare variants. On the other hand, further transcriptomic studies are warranted to improve the understanding of cocaine effects on the brain and the molecular basis of the neuroadaptations that underlie compulsive drug seeking, even after long periods of abstinence. Interestingly, several studies support the idea that genes mediating cocaine’s effect can participate in the predisposition to addiction (e.g., *NFAT5, PLCB1, KCTD20*), highlighting potential therapeutic targets. However, we need more studies that investigate the convergence between altered expression and genetic variation in CUD. We have learned from twin studies and from molecular genetics research that there is a common genetic architecture across different SUDs, but they also reveal some genetic risk factors that are substance-specific. Understanding the weight and nature of each of these components is an important challenge in addiction research. For some drugs of abuse with considerable larger samples sizes, GWAS have started to reveal genetic risk variants in few genes that have been consistently associated and replicated, for instance SNPs in the *ADH1B* gene for alcohol dependence and *CHRNA5* gene for nicotine dependence (recently reviewed [149]). Importantly, a SNP in *CHRNA5* has been associated with differential success rate of treatments in smoking cessation [130]. Despite significant advances in the study of the molecular underpinnings of CUD during the last decade, we are still far from individual genetic prediction to aid prevention, diagnostics or to anticipate disease course and therapeutic response. However, there are promising venues in cocaine research, especially those related to GWAS data: On one hand, we know that PRSs have already been able to identify individuals with risk equivalent to monogenic mutations in several somatic conditions [150, 151] and this is currently being explored also in psychiatric conditions. On the other hand, the relevance of the supporting genetic evidence in the selection of candidates for drug development has been demonstrated across human diseases, increasing by twofold the drug success rate in clinical trials compared to non-genetically selected targets [152, 153]. However, advancing toward more comprehensive pharmacogenetic studies will only be possible once our knowledge on the genetic bases of CUD is wider and more precise.

## Supplementary information

Supplementary Table S1
