# Supplementary material for: Molecular genetics of cocaine use disorders in humans
Source: Mol Psychiatry. 2021 Aug 27;27(1):624–39. doi: 10.1038/s41380-021-01256-1 (PMC8960411; doi:10.1038/s41380-021-01256-1)
Supplement: Supplementary file 1 — Supplementary Table S1 [file 41380_2021_1256_MOESM1_ESM.docx]

**Supplementary Table S1.** Candidate gene association studies on cocaine dependence.

| Studied gene/s | Neurotransmitter system | Significant association | Sample | Ref. |  |
| --- | --- | --- | --- | --- | --- |
| *5-HTT* | Serotonin | no | 156 cases – 82 controls | [1] |  |
| *5-HTT* | Serotonin | no | 105 cases – 44 controls (AA) | [2] |  |
| *5-HTT* | Serotonin | no | 504 cases – 508 controls | [3] |  |
| *ARRB2* | - | no | 336 cases – 656 controls | [4] |  |
| *BChE* | - | yes | 698 cases – 738 controls | [5] |  |
| *CALCYON* | Dopamine | no | EA: 118 cases – 207 controls AA: 90 cases – 46 controls | [6] |  |
| *CARTPT* | - | yes | 348 cases – 256 controls | [7] |  |
| *CHRNA5* | Acetylcholine | yes | 300 cases^&^ – 769 controls | [8] |  |
| *CHRNA5* | Acetylcholine | yes | 260 cases – 244 controls | [9] |  |
| *CHRNA5, CHRNA3*, *CHRNB4* | Acetylcholine | yes | 3,533 cases – 1,483 controls | [10] |  |
| *CNR1* | Endocannabinoid | Yes | 97 cases – 88 controls (AC) | [11] |  |
| *CNR1* | Endocannabinoid | yes | 860 cases – 334 controls | [12] |  |
| *CNR1* | Endocannabinoid | yes (EA) | EA: 734 subjects from 307 families (551 cases) AA: 834 subjects from 299 families (664 cases) | [13] |  |
|  |  | yes (EA) | EA: 175 cases – 403 controls AA: 136 cases – 48 controls |  |  |
| *COMT* | Dopamine | yes | 330 cases – 255 controls | [14] |  |
| *DAT, DBH*, *DRD2* | Dopamine | yes | 169 cases – 169 controls | [15] |  |
| *DRD2* | Dopamine | yes | 53 cases – 265 controls | [16] |  |
| *DRD2*, *SLC6A3/DAT1* | Dopamine | yes | 347 cases – 257 controls | [17] |  |
| *DRD2, DRD3* | Dopamine | no | 730 cases – 782 controls | [18] |  |
| *DRD3* | Dopamine | no | 62 cases – 61 controls | [19] |  |
| *DRIPs* | - | yes | 796 cases – 416 controls | [20] |  |
| Dopaminergic and serotonergic system | Dopamine and serotonin | yes | 432 cases – 482 controls | [21] |  |
| *HTR2B* | Serotonin | yes | 80 cases – 80 controls (AC) | [22] |  |
| *HTR3A/B* | Serotonin | yes (EA) | EA: 454 cases–1,018 controls AA: 458 cases – 397 controls | [23] |  |
| miRNAs binding sites | - | yes | 1,028 cases – 1,406 controls | [24] |  |
| nAChRs or mAChRs | Acetylcholine | yes | 279 cases – 207 controls (AA) | [25] |  |
|  |  |  |  |  |  |
| Neurotransmitters release system | All | yes | 360 cases – 260 controls | [26] |  |
| *NFAT5* | - | yes | 806 cases – 817 controls | [27] |  |
| *NK3R* | - | yes | 219 families | [28] |  |
| *NPY* | - | yes | 1,923 subjects from 219 families (302 cases) | [29] |  |
| *NSF* | All | yes* | 867 cases – 925 controls | [30] |  |
| *NTNG1* | - | Yes | 709 subjects from 146 families (121 cases) | [31] |  |
|  |  |  |  |  |  |
| Opioid receptors | Opioid | yes | 171 cases – 338 controls | [32] |  |
| *OPRD1* | Opioid | yes | EA: 336 cases – 656 controls AA: 503 cases – 503 controls | [33] |  |
| *OPRM1* | Opioid | no | 1,307 cases– 5,168 controls | [34] |  |
|  |  |  |  |  |  |
| *PDYN* | Opioid | Yes | 167 cases – 88 controls (AA) | [35] |  |
| *PDYN* | Opioid | yes | 61 cases – 91 controls | [36] |  |
| *PDYN* | Opioid | yes | EA: 41 cases – 65 controls AA: 128 cases – 76 controls | [37] |  |
| *PDYN* | Opioid | no | EA: 336 cases – 656 controls AA: 1,248 cases– 674 controls | [38] |  |
| *TPH2* | Serotonin | Yes | 299 cases – 208 controls | [39] |  |
|  |  |  |  |  |  |

EA: European-American; AA: African-American; AC: Afro-Caribbean; &crack cocaine dependence. *Copy number variation (CNV). nAChR: nicotinic acetylcholine receptor; mAChR: muscarinic acetylcholine receptor.

1. Patkar AA, Berrettini WH, Hoehe M, Hill KP, Gottheil E, Thornton CC, et al. No association between polymorphisms in the serotonin transporter gene and susceptibility to cocaine dependence among African-American individuals. Psychiatr Genet. 2002;12:161–164.

2. Patkar AA, Berrettini WH, Hoehe M, Thornton CC, Gottheil E, Hill K, et al. Serotonin transporter polymorphisms and measures of impulsivity, aggression, and sensation seeking among African-American cocaine-dependent individuals. Psychiatry Res. 2002;110:103–115.

3. Tristán-Noguero A, Fernàndez-Castillo N, Roncero C, Sánchez-Mora C, Ramos-Quiroga JA, Daigre C, et al. Lack of association between the LPR and VNTR polymorphisms of the serotonin transporter gene and cocaine dependence in a Spanish sample. Psychiatry Res. 2013;210:1287–1289.

4. Ambrose-Lanci LM, Vaswani M, Clarke TK, Zeng A, Lohoff FW, Ferraro TN, et al. Association study of the β-arrestin 2 gene (ARRB2) with opioid and cocaine dependence in a European-American population. Psychiatr Genet. 2012;22:141–145.

5. Negrão AB, Pereira AC, Guindalini C, Santos HC, Messas GP, Laranjeira R, et al. Butyrylcholinesterase genetic variants: Association with cocaine dependence and related phenotypes. PLoS One. 2013;8.

6. Luo X, Kranzler H, Lappalainen J, Rosenheck R, Charney D, Zuo L, et al. CALCYON Gene Variation, Schizophrenia, and Cocaine Dependence. Am J Med Genet - Neuropsychiatr Genet. 2004;125 B:25–30.

7. Lohoff FW, Bloch PJ, Weller AE, Nall AH, Doyle GA, Buono RJ, et al. Genetic variants in the cocaine- and amphetamine-regulated transcript gene (CARTPT) and cocaine dependence. Neurosci Lett. 2008;440:280–283.

8. Aroche AP, Rovaris DL, Grevet EH, Stolf AR, Sanvicente-Vieira B, Kessler FHP, et al. Association of CHRNA5 Gene Variants with Crack Cocaine Addiction. NeuroMolecular Med. 2020;22:384–390.

9. Grucza RA, Wang JC, Stitzel JA, Hinrichs AL, Saccone SF, Saccone NL, et al. A Risk Allele for Nicotine Dependence in CHRNA5 Is a Protective Allele for Cocaine Dependence. Biol Psychiatry. 2008;64:922–929.

10. Sherva R, Kranzler HR, Yu Y, Logue MW, Poling J, Arias AJ, et al. Variation in nicotinic acetylcholine receptor genes is associated with multiple substance dependence phenotypes. Neuropsychopharmacology. 2010;35:1921–1931.

11. Ballon N, Leroy S, Roy C, Bourdel MC, Charles-Nicolas A, Krebs MO, et al. (AAT)n repeat in the cannabinoid receptor gene (CNR1): Association with cocaine addiction in an African-Caribbean population. Pharmacogenomics J. 2006;6:126–130.

12. Clarke TK, Bloch PJ, Ambrose-Lanci LM, Ferraro TN, Berrettini WH, Kampman KM, et al. Further evidence for association of polymorphisms in the CNR1 gene with cocaine addiction: Confirmation in an independent sample and meta-analysis. Addict Biol. 2013;18:702–708.

13. Zuo L, Kranzler HR, Luo X, Yang BZ, Weiss R, Brady K, et al. Interaction between two independent CNR1 variants increases risk for cocaine dependence in european americans: A replication study in family-based sample and population-based sample. Neuropsychopharmacology. 2009;34:1504–1513.

14. Lohoff FW, Weller AE, Bloch PJ, Nall AH, Ferraro TN, Kampman KM, et al. Association between the catechol-O-methyltransferase Val158Met polymorphism and cocaine dependence. Neuropsychopharmacology. 2008;33:3078–3084.

15. Fernàndez-Castillo N, Ribasés M, Roncero C, Casas M, Gonzalvo B, Cormand B. Association study between the DAT1, DBH and DRD2 genes and cocaine dependence in a Spanish sample. Psychiatr Genet. 2010;20:317–320.

16. Noble EP, Blum K, Khalsa ME, Ritchie T, Montgomery A, Wood RC, et al. Allelic association of the D2 dopamine receptor gene with cocaine dependence. Drug Alcohol Depend. 1993;33:271–285.

17. Lohoff FW, Bloch PJ, Hodge R, Nall AH, Ferraro TN, Kampman KM, et al. Association analysis between polymorphisms in the dopamine D2 receptor (DRD2) and dopamine transporter (DAT1) genes with cocaine dependence. Neurosci Lett. 2010;473:87–91.

18. Messas G, Meira-Lima I, Turchi M, Franco O, Guindalini C, Castelo A, et al. Association study of dopamine D2 and D3 receptor gene polymorphisms with cocaine dependence. Psychiatr Genet. 2005;15:171–174.

19. Freimer M, Kranzler H, Satel S, Lacobelle J, Skipsey K, Charney D, et al. No association between D3 dopamine receptor (DRD3) alleles and cocaine dependence. Addict Biol. 1996;1:281–287.

20. Multani PK, Clarke TK, Narasimhan S, Ambrose-Lanci L, Kampman KM, Pettinati HM, et al. Neuronal calcium sensor-1 and cocaine addiction: A genetic association study in African-Americans and European Americans. Neurosci Lett. 2012;531:46–51.

21. Fernàndez-Castillo N, Roncero C, Grau-Lopez L, Barral C, Prat G, Rodriguez-Cintas L, et al. Association study of 37 genes related to serotonin and dopamine neurotransmission and neurotrophic factors in cocaine dependence. Genes, Brain Behav. 2013;12:39–46.

22. Lacoste J, Lamy S, Ramoz N, Ballon N, Jehel L, Maroteaux L, et al. A positive association between a polymorphism in the HTR2B gene and cocaine-crack in a French Afro-Caribbean population. World J Biol Psychiatry. 2019. 2019. https://doi.org/10.1080/15622975.2018.1563721.

23. Yang J, Li MD. Association and interaction analyses of 5-HT3 receptor and serotonin transporter genes with alcohol, cocaine, and nicotine dependence using the SAGE data. Hum Genet. 2014;133:905–918.

24. Cabana-Domínguez J, Roncero C, Pineda-Cirera L, Palma-Álvarez RF, Ros-Cucurull E, Grau-López L, et al. Association of the PLCB1 gene with drug dependence. Sci Rep. 2017;7.

25. Levran O, Randesi M, Peles E, Correa Da Rosa J, Ott J, Rotrosen J, et al. African-specific variability in the acetylcholine muscarinic receptor M4: Association with cocaine and heroin addiction. Pharmacogenomics. 2016;17:995–1003.

26. Fernàndez-Castillo N, Cormand B, Roncero C, Snchez-Mora C, Grau-Lopez L, Gonzalvo B, et al. Candidate pathway association study in cocaine dependence: The control of neurotransmitter release. World J Biol Psychiatry. 2012;13:126–134.

27. Fernàndez-Castillo N, Cabana-Domínguez J, Soriano J, Sànchez-Mora C, Roncero C, Grau-López L, et al. Transcriptomic and genetic studies identify NFAT5 as a candidate gene for cocaine dependence. Transl Psychiatry. 2015;5.

28. Foroud T, Wetherill LF, Kramer J, Tischfield JA, Nurnberger JI, Schuckit MA, et al. The tachykinin receptor 3 is associated with alcohol and cocaine dependence. Alcohol Clin Exp Res. 2008;32:1023–1030.

29. Wetherill L, Schuckit MA, Hesselbrock V, Xuei X, Liang T, Dick DM, et al. Neuropeptide Y receptor genes are associated with alcohol dependence, alcohol withdrawal phenotypes, and cocaine dependence. Alcohol Clin Exp Res. 2008;32:2031–2040.

30. Cabana-Domínguez J, Roncero C, Grau-López L, Rodríguez-Cintas L, Barral C, Abad AC, et al. A Highly Polymorphic Copy Number Variant in the NSF Gene is Associated with Cocaine Dependence. Sci Rep. 2016;6.

31. Kelaï S, Ramoz N, Moalic JM, Noble F, Mechawar N, Imbeaud S, et al. Netrin G1: its downregulation in the nucleus accumbens of cocaine-conditioned mice and genetic association in human cocaine dependence. Addict Biol. 2018;23:448–460.

32. Huiping Zhang ZL. Analyzing Interaction of of μ-, δ- and κ-opioid Receptor Gene Variants on Alcohol or Drug Dependence Using a Pattern Discovery-based Method. J Addict Res Ther. 2013;s7:007.

33. Crist RC, Ambrose-Lanci LM, Vaswani M, Clarke TK, Zeng A, Yuan C, et al. Case-control association analysis of polymorphisms in the delta-opioid receptor, OPRD1, with cocaine and opioid addicted populations. Drug Alcohol Depend. 2013;127:122–128.

34. Schwantes-An TH, Zhang J, Chen LS, Hartz SM, Culverhouse RC, Chen X, et al. Association of the OPRM1 Variant rs1799971 (A118G) with Non-Specific Liability to Substance Dependence in a Collaborative de novo Meta-Analysis of European-Ancestry Cohorts. Behav Genet. 2016;46:151–169.

35. Dahl JP, Weller AE, Kampman KM, Oslin DW, Lohoff FW, Ferraro TN, et al. Confirmation of the association between a polymorphism in the promoter region of the prodynorphin gene and cocaine dependence. Am J Med Genet - Neuropsychiatr Genet. 2005;139 B:106–108.

36. Chen ACH, Steven Laforge K, Ho A, McHugh PF, Kellogg S, Bell K, et al. Potentially functional polymorphism in the promoter region of prodynorphin gene may be associated with protection against cocaine dependence or abuse. Am J Med Genet - Neuropsychiatr Genet. 2002;114:429–435.

37. Yuferov V, Ji F, Nielsen DA, Levran O, Ho A, Morgello S, et al. A functional haplotype implicated in vulnerability to develop cocaine dependence is associated with reduced PDYN expression in human brain. Neuropsychopharmacology. 2009;34:1185–1197.

38. Clarke TK, Ambrose-Lanci L, Ferraro TN, Berrettini WH, Kampman KM, Dackis CA, et al. Genetic association analyses of PDYN polymorphisms with heroin and cocaine addiction. Genes, Brain Behav. 2012;11:415–423.

39. Dahl JP, Cubells JF, Ray R, Weller AE, Lohoff FW, Ferraro TN, et al. Analysis of variations in the tryptophan hydroxylase-2 (TPH2) gene in cocaine dependence. Addict Biol. 2006;11:76–83.
